# KRAS oncogene in non-small cell lung cancer: clinical perspectives on the treatment of an old target

**DOI:** 10.1186/s12943-018-0789-x

**Published:** 2018-02-19

**Authors:** Marta Román, Iosune Baraibar, Inés López, Ernest Nadal, Christian Rolfo, Silvestre Vicent, Ignacio Gil-Bazo

**Affiliations:** 1 Department of Oncology, Clínica Universidad de Navarra, 31008 Pamplona, Spain; 2 Program of Solid Tumors and Biomarkers, Center for Applied Medical Research, Pamplona, Spain; 3 Thoracic Oncology Unit, Department of Medical Oncology, Catalan Institute of Oncology (ICO), L’Hospitalet del Llobregat, Barcelona, Spain; 4 Phase I-Early Clinical Phase I-Early Clinical Trials Unit, Oncology Department, Antwerp University Hospital, Edegem, Belgium; 5 Navarra Health Research Institute (IDISNA), Pamplona, Spain; 6 Centro de Investigación Biomédica en Red de Cáncer (CIBERONC), Madrid, Spain

## Abstract

Lung neoplasms are the leading cause of death by cancer worldwide. Non-small cell lung cancer (NSCLC) constitutes more than 80% of all lung malignancies and the majority of patients present advanced disease at onset. However, in the last decade, multiple oncogenic driver alterations have been discovered and each of them represents a potential therapeutic target. Although *KRAS* mutations are the most frequently oncogene aberrations in lung adenocarcinoma patients, effective therapies targeting KRAS have yet to be developed. Moreover, the role of *KRAS* oncogene in NSCLC remains unclear and its predictive and prognostic impact remains controversial. The study of the underlying biology of *KRAS* in NSCLC patients could help to determine potential candidates to evaluate novel targeted agents and combinations that may allow a tailored treatment for these patients. The aim of this review is to update the current knowledge about *KRAS*-mutated lung adenocarcinoma, including a historical overview, the biology of the molecular pathways involved, the clinical relevance of *KRAS* mutations as a prognostic and predictive marker and the potential therapeutic approaches for a personalized treatment of *KRAS*-mutated NSCLC patients.

## Background

Lung cancer is the most common cancer worldwide both in terms of incidence (1.8 million new cases estimated in 2012) and mortality (1.6 million annual deaths). In fact, lung cancer is the leading cause of death by cancer [1, 2]. Non-small cell lung cancer (NSCLC) comprises about 80% of all lung cancer cases [3]. When patients are diagnosed in early stages of NSCLC the survival rates are relatively higher after surgical resection [4]. However, at the time of diagnosis, the majority of patients have already developed advanced disease and the median survival barely exceeds 18 months from diagnosis [5]. Patients with untreated metastatic NSCLC present an overall survival (OS) rate at one year of only 10%, with a median survival of around 4 to 5 months. Classically, chemotherapy has demonstrated a slight improvement in the survival of patients with advanced NSCLC, reducing symptoms and improving the quality of life. In fact, the effect of the combination of different chemotherapeutic agents with a platinum compound in patients with advanced disease observed no significant differences between the different doublets tested [6]. Those poor results have been significantly improved over the last decade through different therapeutic strategies such as the incorporation of a third antiangiogenic drug to a platinum-based doublet [7], the combination of cisplatin with the antifolate drug pemetrexed [8] and the implementation of pemetrexed maintenance monotherapy after tumor response or stabilization induced by a platinum-based doublet [9].

Also during the last decade, a number of genetic alterations have been described in NSCLC, being *Kristen Rat Sarcoma* viral oncogene (*KRAS*), *Epidermal Growth Factor Receptor* (*EGFR*) and *Anaplastic Lymphoma Kinase* (*ALK*) the most commonly altered oncogenes acting as tumor genomic drivers [10]. The use of targeted therapies in NSCLC individuals with an actionable driver, as *EGFR* and *ALK*, has shown high clinical efficacy in comparison with patients in whom no molecular targets for a personalized therapy are identified [11]. In contrast, regarding *KRAS* oncogene*,* although the KRAS*-*MAPK pathway is downstream of *EGFR* signaling, *KRAS-*mutation driven lung cancers, which are mainly adenocarcinomas, do not respond to EGFR tyrosine kinase inhibitors (TKIs) [12]. Moreover, KRAS activation is one of the signaling pathways involved in resistance to EGFR TKIs and monoclonal antibodies*.* In spite of the EGFR inhibition by TKIs, KRAS activation allows the downstream signaling mediated by EGF [13].

Previous studies have reported that the occurrence of *EGFR* and *KRAS* mutations is strictly mutually exclusive and each of these genetic alterations is associated with specific clinical characteristics such as pathological features, clinical background and prognostic or predictive implications [14, 15]. Nevertheless, recent studies have described concomitant genetic alterations such as *EGFR* or *Echinoderm microtubule-associated protein-like 4* (*EML4*) *ALK* translocation with *KRAS (EGFR/KRAS* or *EML4-ALK/KRAS)*, most of them associated with an acquired mutation after treatment that promotes drug resistance [16, 17]. Tumor heterogeneity according to which different mutations may coexist in different tumor cells or in the same tumor cell could explain this phenomenon of concomitance. *EML4-AKT/KRAS* double alteration represents the most common concomitant genomic aberration and is associated with poor prognosis and resistance to anti-ALK agents. Tumors with concomitant *EGFR/KRAS* mutation usually show the typical histologic patterns and cell characteristics of *EGFR*-mutated tumors and correlates with a better response to EGFR-TKIs therapy [10, 18, 19].

Effective therapies against KRAS have not been developed yet. Indeed, NSCLC adenocarcinoma patients with tumors harboring *KRAS* mutations, that account for 25% of the cases, show a shorter median survival (2.41 years) compared to patients candidates to personalized therapies [20, 21]. Despite all clinical advances regarding personalized therapy, there is still a highly remarkable unmet clinical need since a very well-known and highly prevalent tumor driver mutation in NSCLC patients, such as *KRAS*, still remains refractory to pharmacological inhibition.

## Historical overview

The ability of single-stranded murine sarcoma virus, Kirsten and Harvey, to transform normal mammalian genes into potent oncogenes was discovered over four decades ago [22]. These viral oncogenes were only able to generate rat sarcomas for what they were called *RAS* genes, *KRAS* and *HRAS* alluding to its discoverers [23, 24]. It was not until 1982 when new human sequences homologous to the *HRAS* and *KRAS* oncogenes were identified in human bladder and lung carcinoma cell lines, respectively [25, 26]. The third member of the human *RAS* gene family, designated as *NRAS,* was described in human sarcoma cell lines in 1983 [27, 28].

Mariano Barbacid’s group first established the relationship between *RAS* genes and lung cancer in 1984. They conducted a landmark study which evidenced the presence of an activating mutation of *KRAS* oncogene in a human lung cancer specimen that was not observed in normal tissue of the same patient [29]. Soon after, the prevalence of mutational *KRAS* activation in lung cancers, specifically in NSCLC, was demonstrated [30]. *KRAS* mutations have been found to be almost an exclusive feature of adenocarcinomas and are more frequent in Western populations. Pooled frequencies of *KRAS* mutations range from 6.7% to 40.0% for ever/heavy smokers and from 2.9 to 11.4 for never/light smokers [31]. During the following two decades, studies of RAS focused on its biology and biochemical characteristics both in normal and cancer cells, as well as in the signaling cascade in which RAS is involved [32]. Nevertheless, despite the increase in systematic studies of the *RAS* oncogene, no clinically applicable therapeutic inhibition has proven to be successful for over 30 years. After multiple failed attempts to inhibit RAS either directly or indirectly (downstream effectors and post-transcriptional modifications), ‘The RAS Initiative’ arose (2013), to facilitate connections among RAS researchers to promote new ideas and technologies to bear on RAS. Even so, RAS inhibition and the development of novel therapies remain an unmet clinical need [33–37].

## KRAS biology

**Fig. 1:**
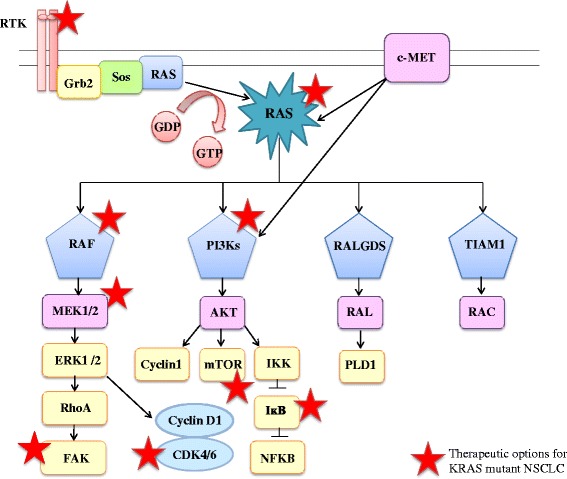
RAS downstream signaling pathways and potential options for therapeutic intervention in lung adenocarcinoma

RAS proteins, including KRAS, are intracellular guanine nucleotide binding proteins (G proteins) which belong to the family of small GTPases. G proteins are composed of a G or catalytic domain, which binds guanine nucleotides and activates signaling, and a C-terminal hypervariable region (HVR) that incorporates farnesyl or prenyl groups (post-transcriptional modifications). These modifications diverge in each isoform because of the sequence variability of the HVR and locate RAS proteins to the cell membrane, where they perform their signaling function [38, 39]. The downstream signaling is regulated by two alternative states of RAS proteins: RAS-GTP (active form) and RAS-GDP (inactive form). RAS-GTP complex activates several downstream signaling effectors such as the canonical Raf-MEK-ERK, the PI3K-AKT-mTOR and RalGDS-RalA/B pathways or the TIAM1-RAC1 pathway (Fig. 1), which control multiple cellular functions including proliferation, apoptosis, motility or survival. These signaling cascades are triggered by coupling of several growth factor receptors like EGFR that favor a constitutive activation of KRAS [33, 40–42] (Fig. 1). The exchange of GDP-GTP is regulated by additional proteins: Guanine nucleotide exchange factors (GEFs) decrease the affinity of RAS proteins for GDP and favor GTP binding that results in RAS activation, while GTPase-activating proteins (GAPs) accelerate the intrinsic GTPase activity to regulate the RAS cycle [43, 44].

Most mutations in *RAS* genes affect exons 2 and 3. These mutations impair the GTPase activity promoting the active GTP-bound state. Generally, the G➔A transition in codons 12 or 13 is dominant in *KRAS* isoform resulting in G12D or G13D mutations, followed by G➔T transversions that produce G12V [45, 46]. The most frequent mutation in *KRAS* mutant NSCLC is G12C (41%). It has been proposed that *KRAS*-mutated tumors behave similarly to the *KRAS*, *EGFR* and *ALK*-native tumors with respect to sites of metastases [47]. However, this may reflect biological heterogeneity, as it has been suggested that the type of point mutation may affect downstream signaling differently, which may translate into different clinical features [48–50]. *G12C* and *G12V* mutations are usually associated with the RalA/B signaling pathway and both of them present shorter progression free survival (PFS). Patients harboring *G12C* mutation are more likely to present bone metastases dissemination, while pleuro-pericardial metastases are more frequent in those with *G12V* mutations. However, *KRAS G12D* mutations preferably activate PI3K and MEK signaling [51, 52]. Furthermore, concurrent mutations of tumor suppressor genes in *KRAS*-mutant adenocarcinoma patients (e.g. *TP53*, *LKB1* or *KEAP1*) should be taken into account because such mutational pattern is related to the control of distinct tumorigenic pathways [53–57]. Thus, tumors initiated with the same oncogenic driver may require different therapeutic approaches. In addition, recent work has established two different groups of *KRAS* mutant NSCLC, *KRAS*-dependent or *KRAS*-independent, according to their requirement for mutant *KRAS* to maintain viability. Gene expression profiles of NSCLC cell lines show a gene expression signature in *KRAS*-dependent cells associated with a well-differentiated epithelial phenotype, whereas *KRAS* independency correlated with an epithelial-mesenchymal transformation (EMT) phenotype. These data suggest that there are specific pathways and activated genes according to the *KRAS* dependency that have an important role in the different cancer phenotypes and their potential treatments [58, 59].

## *KRAS* mutations as a prognostic factor

Although *KRAS* mutations have been classically defined as a negative prognostic factor with more undifferentiated tumors having unfavorable survival rates and disease-free survival compared to *KRAS* wild-type tumors [60], its real clinical significance remains controversial due to heterogeneity amongst studies. Table 1 summarizes the available data of the prognostic value of *KRAS* status in early and advanced NSCLC.

**Table 1:** KRAS status as a prognostic marker in lung cancer

Reference	Type of study	Patients tested for KRAS	Patients by KRAS status		Results (KRAS-mut vs KRAS-wt)
			KRAS-mut	KRAS-wt	
Mascaux et al., 2005 [61]	Pooled analysis	3620 (stage I-IV)	652	2968	HR for OS 1.35 (1.16–1.56), *p* = 0.01
Sheperd et al., 2013 [62]	Pooled analysis	1543 (stage I-III)	300	1246	HR for OS 1.17, 95% CI 0.96 to 1.42, *p* = 0.12
Zer et al., 2016 [63]	Pooled analysis	577 (stage IIIB-IV)	120	457	HR for OS 1.09, 95% CI 0.85–1.41, *p* = 0.48
Pan et al., 2016 [64]	Pooled analysis	13,103 (stage I-IV)	2374	10,729	HR for OS 1.56, 95% CI 1.39–1.76, *p* = 0.00
Svaton et al., 2016 [66]	Individual study	129 (stage IIIB-IV)	39	90	OS: 16.1 months for wt-KRAS and 7.2 for mut-KRAS
					PFS: 2.3 for wt-KRAS and 1.6 for mut-KRAS
Fan et al., 2017 [65]	Pooled analysis	658 (advanced NSCLC)	93	565	HR for PFS 1.83, 95% CI 1.40–2.40, *p* < 0.0001
		693 (advanced NSCLC)	106	587	HR for OS 2.07, 95% CI 1.54–2.78, p < 0.0001

In view of disparity from individual studies, several meta-analysis have been conducted. A meta-analysis of 28 studies and 3620 patients demonstrated the negative prognosis of *KRAS* in lung adenocarcinomas, but not in squamous-cell carcinoma histology tumors [61]. However, it must be noted that *EGFR*-mutations, which are well-known to have a better prognosis and be, in general, mutually exclusive, were not taken into account, leading to a possible overestimation in the control arm.

In 2013, a different meta-analysis conducted by the Lung Adjuvant Cisplatin Evaluation (LACE)-BIO collaborative group that included data from four clinical trials (ANITA, IALT, JBR.10, and CALGB- 9633) was published [62]. No significant differences in the prognostic value neither in the overall group nor when patients were divided by histology were found.

In 2015, pooled data from four trials of EGFR TKIs versus placebo (National Cancer Institute of Canada Clinical Trials Group [NCIC CTG] trial BR.21, TOPICAL, NCIC CTG trial BR.26, and NCIC CTG trial BR.19) including known *KRAS* status for 1362 of 2624 patients, found no statistically significant differences in OS in the placebo arms between patients harboring *KRAS* mutations and those with *KRAS* native status [63].

However, another meta-analysis of 41 studies has described the negative prognostic value of *KRAS* mutations, showing a worse OS and disease-free survival (DFS) when mutations are present [64]. Furthermore, a recently published meta-analysis exploring the prognostic value of *KRAS* mutations in circulating tumor DNA indicated a worse PFS and OS in patients harboring *KRAS* mutated genotypes [65].

Concerning *KRAS* mutation subtypes, retrospective studies have shown that patients with early stage and advanced NSCLC harboring G12C *KRAS* mutations had significantly shorter OS compared to other *KRAS* mutations [66, 67]. In this cohort, there were no differences between both groups for PFS.

In addition to the prognostic impact of the presence of *KRAS* mutations, concurrent mutations in other genes may have an added prognostic value. On the one hand, *EML4-ALK* fusion has been proven to be associated with poor prognosis when *KRAS* mutations are also co-present [19]. On the other hand, *KRAS* mutated NSCLC patients harboring mutations in the tumor suppressor genes *STK11/LKB1* or *CDKN2A* show a worse prognosis than those with *TP53* mutations [53].

## *K*RAS mutations as a predictive factor

### Predictive value of *KRAS* mutations for response to chemotherapy

Most patients with advanced lung cancer receive treatment with chemotherapy regimens based on platinum. *KRAS* status has been studied in this clinical setting as a biomarker to predict the expected clinical outcome to chemotherapy. However, data to support the predictive value of *KRAS* mutations in this specific clinical scenario are limited. Table 2 summarizes the predictive value of *KRAS* mutations for response to therapies.

**Table 2:** KRAS status as a predictive marker in lung cancer

Reference	Patients tested for KRAS	Patients by KRAS status		Treatment arm	Endpoint	KRAS-mut	KRAS-wt
		KRAS-mut	KRAS-wt				
Rodenhius et al., 1997 [68]	62 (stage III-IV)	16	46	Carboplatin + ifosfamide + etoposide	ORR (%)	19	26
					PFS (months)	4	5
					OS (months)	8	9
Schiller et al., 2001 [69]	184 (stage II-IIIA)	44	140	Cisplatin + etoposide	OS (months)	24.7	42
Eberhard et al., 2005 [70]	133 (advanced stage)	25	108	Carboplatin + paclitaxel + erlotinib	ORR (%)	23	26
					PFS (months)	6.0	5.4
					OS (months)	13.5	11.3
Tsao et al., 2007 [71]	210 (stage Ib-II)	46	164	Vinorelbine + cisplatin	OS (months)	6.4	NR
Mao et al., 2010 [76]	1470 (stage NS)	231	1239	EGFR TKI	ORR (%)	3	26
Khambata-Ford et al., 2010 [82]	202 (stage IIIB, IV)	35	167	Taxane + carboplatin + cetuximab	ORR (%)	30.8	32.9
					PFS (months)	5.6	5.1
					OS (months)	16.8	9.7
O’Byrne KJ et al., 2011 [83]	395 (stage IIIB, IV)	75	320	Cisplatin + vinorelbine + cetuximab	ORR (%)	36.8	37.3
					PFS (months)	5.4	4.4
					OS (months)	8.9	11.4
Ludovini V et al., 2011 [79]	166 (stage III, IV)	11	151	EGFR TKI	ORR (%)	0	35.7
					PFS (months)	2.7	5.6
					OS (months)	19.3	28.6
Metro et al., 2012 [81]	67 (stage IIIB-IV)	18	49	EGFR TKI (gefitinib or erlotinib)	PFS (months)	1.6	3.0
					OS (months)	6.0	21.0
Fiala O et al., 2013 [80]	448 (stage IIIB, IV)	138 (G12C mutation: 38)	410	EGFR TKI	PFS (weeks)	4.3 (G12C) vs 9.0 (non-G12C)	
					OS (weeks)	12.1 (G12C) vs 9.3 (non-G12C)	
Zer et al., 2016 [63]	785 (stage IIIB-IV)	155	630	EGFR TKI (pooled analysis)	OS (months)	4.5	6.0
Hames ML et al., 2016 [73]	150 (stage IV)	80	70	Conventional chemotherapy	PFS (months)	4.5	5.7
					OS (months)	8.8	13.5
Dong ZY et al., 2017 [84]	34 (not specified)	8	26	Pembrolizumab	ORR (%)	25	6.6
	20 (not specified)	5	15	Pembrolizumab or nivolumab	PFS (months)	14.7	3.5

In 1997, Rodenhuis et al. assessed the influence of *KRAS* mutations on the response to chemotherapy (carboplatin, ifosfamide and etoposide) in the metastatic setting [68]. Response rate and median OS did not differ according to *KRAS* status. Neither Schiller et al. found differences in OS when assessing the potential benefit of postsurgical chemotherapy (cisplatin and etoposide) added to thoracic radiation in patients with stage II and IIIA NSCLC according to *KRAS* status [69]. In 2005, results from the phase III, Tarceva Responses in Conjunction with Paclitaxel and Carboplatin (TRIBUTE) trial in advanced NSCLC comparing first-line carboplatin/paclitaxel plus erlotinib or placebo were published [70]. Response rate, median time to progression and median OS did not differ either between mutant and wild-type tumors.

The results of the JBR10 trial, which studied the effect of postoperative chemotherapy (vinorelbin and cisplatin) in patients with resected stage IB or II NSCLC were reported in 2010 [71]. Significant benefit from chemotherapy was reported in *KRAS* wild-type patients receiving chemotherapy, whereas there were no differences in the *KRAS* mutant group. The *p* value for the interaction analysis was 0.29, showing no statistical significance, meaning that *KRAS* status has no value as a predictor of survival in patients treated with adjuvant chemotherapy.

The phase III IFCT-0002 trial compared two chemotherapy regimens (carboplatin and paclitaxel vs cisplatin and gemcitabine) and two sequences of chemotherapy (neoadjuvant vs perioperative) in stage I and II NSCLC [72]. Univariate analyses showed that *KRAS* status was associated with response to chemotherapy. However, this association was not significant in the multivariate analysis.

Nonetheless, in a recently published retrospective analysis of a cohort of patients with advanced NSCLC, patients harboring *KRAS* activating mutations exhibited a lower proportion of responses to cytotoxic chemotherapy and decreased survival compared to patients harboring native *KRAS* [73]. Co-mutation of *TP53* and *KRAS* has also been studied, showing worse OS in patients harboring co-mutation versus double wild type tumors [74]. The induction of a different sensitivity pattern depending on the specific *KRAS* mutation has been studied in the preclinical scenario by generation of NSCLC cell lines overexpressing the three most common amino acid substitutions (G12C, G12V and G12D) leading to the *KRAS*-mutated proteins [75]. Whereas the expression of G12V shows resistance to paclitaxel and sensitivity to sorafenib, the expression of G12C is related to reduced response to cisplatin and sensitivity to pemetrexed and paclitaxel. G12V mutations resulted in resistance to pemetrexed and sensitivity to cisplatin. There was no correlation between *KRAS* mutations and response to gemcitabine and EGFR inhibitors. Overall, studies published to date about the predictive value of *KRAS* mutations show no consistent data and therefore, *KRAS* status should not be used as a predictive factor to select patients for specific chemotherapy regimens.

### Predictive value of *KRAS* mutations for response to targeted therapy

KRAS has been widely studied as a predictive biomarker for response to targeted agents, in clinical trials involving anti-EGFR therapies in NSCLC. Two meta-analyses evaluating erlotinib and gefitinib have suggested a negative predictive value of KRAS-mutated tumors harboring *EGFR* activating mutations treated with EGFR TKIs [76, 77]. However, these data are confused by the fact that *KRAS* mutations result in persistent activation of the EGFR-RAS-RAF-ERK-MEK pathway, even when EGFR is inhibited. When excluding *EGFR*-mutated tumors from the analyses, data are controversial. While some investigations have not found statistically significant differences in terms of overall response rate (ORR) or PFS according to *KRAS* mutation status [78], others have found *KRAS* to be a negative predictor for EGFR-TKI treatment [79].

A potential predictive value of particular *KRAS* mutation subtypes has also been postulated. Patients harboring the dominant G12C *KRAS* mutation had shorter PFS and OS than those with non-G12C *KRAS* mutations in a subgroup of 38 patients harboring a mutated *KRAS* gene and wild-type *EGFR* gene who were treated with erlotinib or genitinib [80]. Patients harboring other *KRAS* mutations than the G12C type showed similar PFS and OS to patients harboring the wild-type-*KRAS*, wild-type *EGFR* genotype. Codon 13 mutations also seem to confer a worse outcome than codon 12 mutations [81].

Another recently published pooled analysis including four trials testing EGFR TKIs documented an OS benefit among patients with tumors showing the G12D/S mutation, whereas treatment with EGFR TKIs resulted harmful for those with the G12V mutations [63].

In contrast to *KRAS* mutant colorectal cancer, where *KRAS* mutations are predictive of poor response to anti-EGFR monoclonal antibodies, cetuximab and panitumumab, BMS099 and FLEX clinical trials demonstrated no statistically significant association between *KRAS* status and ORR, PFS, or OS when cetuximab was added to platinum-based chemotherapy in patients with advanced NSCLC [82, 83].

Furthermore, *KRAS* mutations have also been described as potential biomarkers for response to immune checkpoint inhibitors through alteration of a group of genes involved in cell cycle regulating, DNA replication and damage repair. A remarkable clinical benefit to PD-1 inhibitors has been showed in *TP53*, *KRAS* or *TP53/KRAS* patients [84]. These evidences are related to the positive correlation between *KRAS* mutation and PD-L1 expression in lung adenocarcinoma, which represents the innate immune resistance. PD-L1 seems to be up-regulated in models of NSCLC with mutation in the *KRAS* oncogene through p-ERK, hence PD-1 inhibitors as pembrolizumab or ERK inhibitor might recover the tumor immunity of CD3+ T cells, that normally become apoptotic and promote the immune scape [85].

## Targeted therapies for *KRAS*-mutant lung cancer

To date, no efforts at targeting KRAS have proven to be successful (Table 3). Moreover, different strategies for direct inhibition of specific *KRAS*-mutated proteins using several strategies such as an irreversible allosteric inhibitor of G12C RAS to prevent GTP-KRAS formation [37], compounds that target the guanine nucleotide binding pocket (SML-8-73-1) [86] or allele-specific inhibitors (ARS-853) [87, 88] have been reported. Although these compounds showed great specificity towards inhibition of mutant *KRAS* tumors in vitro and provide proof-of-concept of direct KRAS inhibition, long-term efficacy as well as toxicity remains as standing hurdles. Therefore, currently non-specific chemotherapy with conventional cytotoxic drugs remains as the standard treatment for *KRAS*-driven lung cancers.

**Table 3:** Clinical trials investigated in non-small cell lung cancer targeting KRAS pathway

Target	Drug/drug combination	Date	Phase	Patients	Line	KRAS status	Primary endpoint	RR, %	PFS (months)	OS (months)	NCT
Farnesyl transferase	Tipifarnib [91]	2003	II	44	1	Unknown	ORR	0	2.7	7.7	NCT00005989
Farnesyl transferase	CI-1040 [102]	2004	I	67	≥1	Unknown	ORR	0	4.4	NA	NCT00033384
C-Raf	PD-0325901 [105]	2010	II	34	≥1	Unknown	ORR	0	1.8	7.8	NCT00174369
C-Raf	Selumetinib/pemetrexed [107]	2010	II	84	2 or 3	Unknown	Disease progression event	5 vs 4	2.2 vs 3	NA	NCT00372788
Mek	Salirasib [92]	2011	II	33	All lines	Mut	Rate of nonprogression at 10 weeks	0	TTP: 2 (1st line), 1 (2nd line)	Not reached (1st line), 15 (2nd line)	NCT00531401
Mek	Tivantinib + erlotinib/placebo + erlotinib [120]	2011	II	167	> 1	Mut, wt, unknown	PFS	10 vs 7	3.8 vs 2.3	8.5 vs 6.9	NCT00777309
Mek	RO5126766 [103]	2012	I	52	All lines	Unknown	Safety	6.6	NA	NA	NCT00777309
Mek	Cobimetinib + pictilisib [129]	2012	Ib	78	NS	Unknown	Safety	14	NA	NA	NCT00996892
Mek	Ridaforolimus [126]	2012	II	79	> 1	Mut	PFS	1	4	18	NCT00818675
Mek	Tivantinib + erlotinib/placebo + erlotinib [121]	2012	III	1048	2 or 3	Mut, wt, unknown	OS	NS	3.6 vs 1.9	8.5 vs 7.8	NCT01244191
Mek	RO5126766 [104]	2013	I	12	> 1	Unknown	Safety	0	NA	NA	–
Mek	Trametinib + docetaxel [113]	2013	I/Ib	46	> 1	Mut, wt	Safety	17 (KRAS-mut: 17)	NA	NA	NCT01192165
Proteasome	Trametinib + pemetrexed [116]	2013	I/Ib	42	> 1	Mut, wt	Safety	14.3 (KRAS-mut: 15)	NA	NA	NCT01192165
Mek	Trametinib + gemcitabine (2014)	2013	Ib	31	All lines	Unknown	Safety	30	NA	NA	NCT01428427
Met	Pimasertib + voxtalisib [130]	2013	Ib	53	NS	NS	Safety	7	NA	NA	NCT01390818
Mek	Sorafenib [98]	2013	II	59	> 1	Mut	DCR at 6 weeks	10.5	2.3	5.3	NCT00064350
Mek	Selumetinib + docetaxel/placebo + docetaxel [109]	2013	II	87	2	Mut	OS	37 vs 0	5.3 vs 2.1	9.4 vs 5.2	NCT00890825
Met	Onartuzumab + erlotinib/placebo + erlotinib [122]	2013	II	137	≥2	Mut, wt, unknown	PFS	5.8 vs 4.4	2.2 vs 2.6	8.9 vs 7.4 (KRAS-mut: 10.4 vs 7.7)	NCT00854308
Met	Ganetespib [137]	2013	II	99	> 1	Mut, wt	PFS at 16 weeks	KRAS-mut: 0	KRAS-mut: 1.9	KRAS-mut: 11	NCT01031225
mTOR	Copanlisib + refametinib [131]	2014	Ib	49	NS	Mut, wt, unknown	Safety	2.2	NA	NA	NCT01392521
Mek, PI3k	Alpelisib + binimetinib [132]	2014	Ib	58	NS	Mut	Safety	8.6	NA	NA	NCT01449058
Mek, PI3k	Trametinib/docetaxel [113]	2015	II	129	2	Mut	PFS	12 vs 12	3 vs 2.75	2 vs NR	NCT01362296
Mek, PI3k	Bortezomib [119]	2015	II	16	≥2	Mut	ORR	6.6	1	13	NCT01833143
Fak	Defactinib [134]	2015	II	55	≥2	Mut	PFS at 12 weeks	1.8	NA	NA	NCT01951690
Hsp90	Ganetespib + docetaxel/Docetaxel [138]	2015	II	385	2	Mut, wt	PFS	NA	KRAS-mut: 3.9 vs 3.0	KRAS-mut: 7.6 vs 6.4	NCT01348126
Mek, PI3k, mTOR	Sorafenib/placebo [99]	2015	III	706	3 or 4	Mut, wt	OS	4.9 vs 0.9 (KRAS-mut: 2.9 vs 0)	2.8 vs 1.4 (KRAS-mut: 2.6 vs 1.7)	8.2 vs 8.3 (KRAS-mut: 6.4 vs 5.1)	NCT00863746
Mek	Selumentinib + erlotinib/placebo + erlotinib [112]	2016	II	89	2 or 3	Mut, wt	PFS, ORR	10 vs 0 (mut)	2.3 vs 4 (mut)	21.8 vs 10.5 (mut)	NCT01229150
Host immunity	Paclitaxel + carboplatin + reolysin [139]	2016	II	37	≥1	Mut, wt	ORR	31	12	4	NCT 00861627
Hsp90	Selumetinib + docetaxel/placebo + docetaxel [111]	2017	III	505	2	Mut	PFS	20.1 vs 13.7	3.9 vs 2.8	8.7 vs 7.9	NCT01933932

Indirect strategies for targeting KRAS pathway have been investigated as well. Considering that, *KRAS* mutations result in activation of the cascade RAF-MEK-ERK and NF-kB, potential targeted therapies for *KRAS*-mutant lung cancers have focused on inhibiting downstream effectors of this signaling pathway (Fig. 1). Constitutive activation of KRAS leads to the persistent stimulation of downstream signaling pathways that promote tumorigenesis and maintains the oncogenic phenotype, including the PI3K/AKT/mTOR cascade, RHO-FAK pathways and overexpression of MET receptor. Inhibition of these cascades has been tested in preclinical and clinical models.

### Farnesyl transfeRASe inhibitors

Given that KRAS ought to be farnesylated to localize in cell membrane, strategies to prevent these post-translational modification have been developed. Despite promising in vitro and in vivo results in preclinical models demonstrated that farnesyl transferase inhibitors (FTI) such as tipifarnib (R115777) or salirasib could prevent the development of lung tumors [89, 90], phase II trials using FTI failed to show clinical activity.

Tipifarnib was tested in 44 patients with advanced NSCLC [91]. Although in vivo activity of tipifarnib in patient tissues was documented, it only translated into a modest clinical activity, since no objective complete or partial responses were seen and only seven patients experienced disease stabilization for at least 6 months.

In a phase II trial testing salirasib, among the 33 patients with advanced lung adenocarcinoma enrolled, 30 showed tumors harboring *KRAS* mutations (23 previously treated patients and 7 treatment-naïve individuals) [92]. Among the 23 previously treated patients, 30.4% (7/23) showed stable disease at 10 weeks with a median time to progression of 2 months. Median time to progression to first line salirasib was 1 month, with a 40% stable disease rate.

### BRaf inhibitors

Clinical attempts to block downstream KRAS signaling pathways through Raf inhibition also yielded disappointing results.

BRaf inhibitors used against *BRaf*-mutated melanomas, such as vemurafenib or dabrafenib, are unlikely to prove any meaningful clinical effect as targeted agents in *KRAS*-mutated NSCLC, since *KRAS* and *BRaf* activating mutations are mutually exclusive [93]. More importantly, inhibition of activating BRaf mutations in mutant *KRAS* tumors induces Erk phosphorylation in a Craf-dependent manner to promote tumorigenesis, in what is known as the MAPK paradox [94, 95], thus discouraging the use of BRaf inhibitors in oncogenic *KRAS* tumors.

Alternatives include inhibition of other Raf members critical for mutant *KRAS*-driven NSCLC [96]. Sorafenib, an oral multi-tyrosine kinase inhibitor that targets Raf and related transmembrane receptors, was seen to induce CRaf depletion and, secondarily, inhibit cell growth and induce G1 arrest in NSCLC *KRAS-*mutant cells [97]. However, clinical attempts to inhibit Raf using sorafenib have been disappointing. A phase II clinical trial testing sorafenib in patients with advanced NSCLC who had progressed to at least one platinum-containing regimen showed disease control in 53% of the 57 patients enrolled, but only 9% experienced a documented radiologic response to the treatment [98]. In the MISSION trial, a phase III multicenter, placebo-controlled study that tested sorafenib in patients with relapsed or refractory non-squamous NSCLC after 2 or 3 previous chemotherapy regimens, PFS but not OS was significantly longer in both mutated and wild type-*KRAS* patients [99]. In the BATTLE trial (Biomarker-integrated Approaches of Targeted Therapy for Lung Cancer Elimination), sorafenib achieved a better disease control rate in mutant-*KRAS* patients (61% versus 32%) compared with the combined other treatments (erlotinib, vandetanib or erlotinib) in chemorephractory NSCLC patients. However, these differences were not statistically significant (*p* = 0.11) [100].

### MEK inhibitors

Several agents targeting MEK, which acts downstream of KRAS (Fig. 1), to suppress signaling through the mitogen-activated protein kinase (MAPK) cascade seem to have greater antitumor activity in tumors harboring *RAS* or *BRaf* mutations [101], whose proliferation and survival rely on the activation of the RAF-MEK-ERK pathway.

Despite their preclinical activity, first clinical trials using MEK inhibitors as CI-1040 [102], RO5126766 [103, 104] and PD-0325901 [105] in non-selected populations of different tumors types harboring *KRAS* mutations yielded disappointing results, more likely due to activation of resistance mechanisms through compensatory signaling effectors [106].

Based on their preclinical activity, clinical trials testing more recently developed MEK inhibitors, such as selumetinib and trametinib have been also conducted.

A phase II trial comparing single agent selumetinib (AZD6244 or ARRY-142886) versus pemetrexed in previously treated patients with advanced NSCLC and unreported *KRAS* status showed no significant clinical benefit in terms of RR or median PFS [107]. Preclinical data demonstrated that AZD6244 has potential to inhibit tumor proliferation, induce differentiation and apoptosis activity in *KRAS*-mutant xenograft models and that antitumor efficacy was improved by combining with cytotoxic drugs as docetaxel [108]. Based on this, another phase II trial testing the synergistic effect of adding selumetinib to docetaxel in previously treated *KRAS*-mutant patients was conducted [109]. It showed clinical benefit in PFS and ORR, whereas no improvement in OS and more toxicity was recorded in the selumetinib-docetaxel arm. A subgroup analysis of *KRAS*-mutations subtypes documented that patients harboring G12V and G12C mutations seemed to experience higher RR and PFS was longer for the combination arm [110]. A non-significant trend toward longer survival was seen in the G12C mutation subgroup. Clinical trials with a G12V and G12C mutations selected population have not been performed yet. The phase III trial did not confirmed the efficacy data, with no improvement in RR, PFS and OS in the combination arm [111].

Combination of selumetinib and erlotinib in NSCLC patients who had progressed to one or prior regimens was also studied in a phase II trial, where patients with neoplasms harboring *KRAS* mutations were randomized to selumetinib in monotherapy or to the combination arm, whereas patients with *KRAS* wild-type tumors were randomized to either erlotinib or the combination therapy. In *KRAS* mutant NSCLC patients, no responses were seen in the monotherapy cohort, and the combination therapy failed to show significant improvement in PFS or ORR and caused more adverse events [112].

Trametinib has also been tested in clinical trials, with similar results. In a phase II trial, the use of trametinib in monotherapy compared to docetaxel in previously treated *KRAS* driven NSCLC showed similar PFS and RR in both groups [113]. Other combinations of trametinib with gemcitabine, pemetrexed and docetaxel have been tested in phase Ib clinical trials concluding that further investigations are warranted in order to demonstrate their clinical activity [114–117].

Phase I and II clinical trials using MEK inhibitors in combination with other therapies are still recruiting patients or under evaluation in *KRAS*-mutated NSCLC (NCT02964689 evaluating binimetinib in addition to standard chemotherapy; NCT01859026 studying MEK162 in combination with erlotinib; NCT02022982 investigating palbociclib and PD-03259019).

### NF-kB pathway

Preclinical studies have provided evidence of the dependence of NF-kB pathway of tumor cells harboring *KRAS* mutations for their viability. Activity modulation of NF-kB by preventing the degradation of NF-kB inhibitor (IkB) using proteasome inhibitors or knocking down TKB1 (an IkB kinase that enhances NK-kB) translates into apoptosis of *KRAS*-mutated cells [118].

According to these data, a phase II single-institution clinical trial (NCT01833143) testing subcutaneous bortezomib, a downregulator of the NF-kB pathway, in patients with advanced NSCLC harboring *KRAS* G12D mutation or no past smoking history is ongoing at Memorial Sloan-Kettering Cancer Center. Partial results were presented at 2015 ASCO Annual Meeting, showing modest antineoplastic activity. Indeed, regarding the RR, one partial response and six stabilizations of disease of a total of 16 patients enrolled where reported [119].

### MET inhibitors

A phase II trial comparing erlotinib alone or in combination with the MET inhibitor tivantinib (ARQ 197) in previously treated EGFR TKI-naïve unselected advanced NSCLC failed to demonstrate clinical benefit in PFS in this cohort [120]. Nevertheless, exploratory analyses demonstrated significant differences in PFS in the subgroup of patients harboring *KRAS* mutations. A phase III clinical trial named MARQUEE, compared erlotinib and tivantinib with erlotinib and placebo in patients with locally advanced or metastatic, nonsquamous NSCLC and stratified the cohort by *KRA*S and *EGFR* status [121]. Despite an improvement in PFS was shown, the Data Monitoring Committee closed the study prematurely, because the data had crossed the futility boundary. Subgroup results concerning the cohort of *KRAS*-mutant patients have not been reported yet.

Onartuzumab, a monoclonal antibody that targets MET receptor, has also been tested in combination with erlotinib in molecularly unselected recurrent NSCLC patients [122]. In this phase II clinical trial, although there were no statistically significant differences in RR, PFS or OS between both arms (onartuzumab plus erlotinib vs. o placebo plus erlotinib), significant differences in PFS and OS between groups were observed in favor of the MET-positive group. In an exploratory subgroup analysis concerning potential predictive biomarkers, no responses were observed in the group of patients harboring *KRAS* mutations [123].

### Targeting PI3K-AKT-mTOR pathway

It has been demonstrated that *KRAS* mutations can coexist with PI3K activation in tumors at the same time [124]. However, based on preclinical data, monotherapy with PI3K inhibitors seems to be insufficient in tumors harboring *KRAS* mutations as the RAF-MEK-ERK pathway hijacks tumor growth through compensatory mechanisms.

The blockade of the pathway using mTOR inhibitors, arresting tumor cells in G1 phase, has also been tested [125]. Ridaforolimus, an mTOR inhibitor, has been tested in a phase II trial in advanced NSCLC harboring *KRAS* mutations [126]. Although PFS was significantly improved, RR was only 1% in the ridaforolimus arm in compared with the placebo arm and no significant differences in OS were identified.

In order to block KRAS signaling completely, preclinical studies had suggested dual inhibition of PI3K/AKT/mTOR and BRAF/MEK/ERK pathways as an effective approach [127]. This modality has also been studied in the clinic [128]. Phase I trials in unselected advanced solid tumors using PI3K combined with either MEK or mTOR inhibitors are now under evaluation [129–132]. Although no preliminary data in *KRAS*-mutated population have been reported yet, important toxic effects could be anticipated given the importance of these two signaling pathways in normal cells homeostasis.

### Targeting FAK

The RHOA-FAK pathway, involved in cell migration, has also proved to play an important role in some *KRAS*-mutated tumors, in which the mutation of *KRAS* added to inactivation of the tumor suppressor genes INK4a/ARF/p16, leads to hyperactivation of the GTPase RHOA by MEK1/2 and ERK1/2 [133]. Despite the absence of specific drugs targeting RHOA, FAK inhibitors have been developed. Defactinib, a FAK inhibitor V2–6063, is being tested in heavily pretreated patients with *KRAS*-mutant NSCLC in an ongoing clinical trial. Partial results were presented at the 16th World Conference on Lung Cancer in 2015, showing a 12-weeks PFS of 36%, but efficacy did not appear to correlate with INK4a/ARF/p16 status [134].

### Targeting HSP90

Inhibition of heat shock proteins has been tested as another potential therapeutic strategy in the *KRAS*-mutated NSCLC scenario. The molecular chaperone Hsp90 is required for proteins’ stability and maturation and protection from proteasomal degradation. Many of these proteins are signaling transduction proteins, such as EGFR, RAF, AKT or products of mutated overexpressed oncogenes that maintain the oncogenic phenotype. Therefore, inhibition of heat shock proteins results in blockade of multiple oncogenic signaling pathways in tumor cells [135]. Treatment with ganetespib, an Hsp90 inhibitor, of *KRAS*-mutated cells resulted in decrease levels of EGFR, MET and CRAF, leading to inactivation of the RAS/RAF/MEK/ERK and PI3K/AKT pathways resulting consequently in apoptosis [136].

Clinical trials using ganetespib in monotherapy or in combination with other drugs, such as chemotherapy, MEK inhibitors, PI3K/mTOR inhibitors or mTOR inhibitors have been tested as well, with disappointing results in the *KRAS*-mutated setting [136–138].

### Other strategies

Additional therapeutic strategies for mutant *KRAS* NSCLC such as reovirus type 3 [139], docetaxel nanoparticles (NCT02283320) or abemaciclib (a cell cycle inhibitor selective for the cyclin-dependent kinases CDK4 and CDK6) [140] are currently under development. Preliminary data analysis of a phase III trial testing abemaciclib in monoteraphy in *KRAS*-mutated advanced NSCLC did not meet its primary endpoint of OS (not published yet) .

Preclinical data raises interest in some of these therapies, as CDK4 had been identified as a synthetic lethal partner with KRAS oncogene in a study that shows genetic and pharmacological evidence demonstrating the role of CDK4 in proliferation of *KRAS*-mutant lung cells [141].

In addition, with the advent of checkpoint inhibitors, and given the high burden of neo-antigens associated to *KRAS*-mutated NSCLC, the use of immunotherapy in *KRAS*-mutated NSCLC appears as a novel therapeutic option with promising results. In fact, *KRAS* mutations, in conjunction with *TP53* mutations, have been recently proposed as biomarkers to predict clinical benefit from PD-1/PD-L1 blockade [84]. Moreover, the ineffectiveness of immunotherapy in *KRAS/LKB1* patients has been described and associated with a marked increase in inflammatory cytokines that recruit neutrophils and block T cells [142]. Complementary to the previous therapeutic strategies, many preclinical investigations have been carried out or are under way with the aim of discovering potential therapeutic targets for the treatment of *KRAS-*activated NSCLC adenocarcinoma patients. Among them, loss-of-functions screens have spearheaded the identification of KRAS dependencies or synthetic lethal interactions in the last decade. These have unveiled molecular targets potentially amenable to therapeutic intervention such as PLK1 [143], TBK1 [118]], BCL-XL [144], FAS [145] and XPO1 [146]. Other approaches have focused on gene-expression analyses of early events in oncogenesis, building upon the premise that inhibition of such events could attenuate tumor growth and relapse [147]. These studies led to the identification of the kinase receptor DDR1 [148] and the transcription factor FOSL1 [149] as KRAS vulnerabilities in mutant *KRAS* tumors. Notably, these studies provided the rationale for combinatorial approaches involving either inhibition of DDR1 and Notch signaling [148] or inhibition of the FOSL1 target AURKA and MEK [148], both of which blocked tumor initiation and progression as well as induced tumor regression. Additionally, chemical screens have unveiled further options to treat mutant *KRAS* cancers using combinatorial strategies, which included the combination of IGFR1 and MEK inhibitors [150], TNKR and MEK inhibitors [151], or PLK1 and ROCK inhibitors [152]. Lastly, diverse research lines are currently open in this field, leading to the identification of promising unconventional therapeutic targets such as miR-1298 that inhibits tumor growth in *KRAS*-driven tumors [153] or the Inhibitor of Differention-1 (Id1) [154] that may have chemopreventive and therapeutic efficacy in *KRAS*-mutated lung tumors.

## Conclusions

In conclusion, agents targeting driver oncogenic mutations in the advanced NSCLC setting have already changed the treatment paradigm. Given the high incidence of *KRAS* mutations in patients with NSCLC, this is a promising therapeutic target. However, KRAS is a heterogeneous entity and other coexisting alterations may be crucial for its role and biologic impact. Even though attempts to target KRAS pathway have shed little light so far, new molecules or new therapeutic strategies may revolutionize outcomes in patients with *KRAS*-driven NSCLC in the near future. Further investigations to better understand the pathways involved, to identify possible synthetic lethal partners and for a better patient selection are needed.
